# A novel magnet-actuated droplet manipulation platform using a floating ferrofluid film

**DOI:** 10.1038/s41598-017-15964-8

**Published:** 2017-11-16

**Authors:** Chao Yang, Gang Li

**Affiliations:** Defense Key Disciplines Lab of Novel Micro-Nano Devices and System Technology, Key Laboratory of Optoelectronic Technology and Systems, Ministry of Education, Chongqing University, Chongqing, 400044 China

## Abstract

In this paper, we propose a novel but versatile magnet-actuated platform for droplet manipulation, which uses a ferrofluid film floating on a liquid surface as magnetic actuator. In contrast to the traditional magnetic droplet manipulation, this platform can handle droplets without magnetically functionalizing them. Due to the immiscibility of the oil-based ferrofluid and water, the droplets desired to be manipulated can stably rest on the surface of the floating ferrofluid film (FFF) under the action of surface tension, thereby offering possibilities for magnetically-driven droplet manipulations. Such a floating, magnetically responsive liquid film not only offers an open surface for active 2D droplet manipulation, but also enables complex droplet manipulations in 3D space. Using FFF, we demonstrate a “full-space” droplet manipulation, including droplet transport/coalescence above FFF (*i.e*. in air), droplet transport/coalescence on FFF and droplet encapsulation/release under FFF (*i.e*. in liquid). Furthermore, we investigated the effects of the magnetic field intensity, the ferrofluid concentration, the droplet volume, and the FFF thickness on droplet kinematics. By finely tuning these operating conditions, the FFF strategy can enjoy more operational latitude than traditional droplet systems, thus allowing more versatile liquid handling.

## Introduction

Along with the development of microfluidic technologies, techniques have been developed for manoeuvring the droplets on a planar surface for a broad range of applications from lab-on-a-chip devices for biological and chemical analyses to bioinspired functional surfaces. To date, various approaches have been developed for manipulating droplets, including electrowetting-on-dielectric (EWOD)^1–5^, surface acoustic wave^6–9^, pneumatic actuation^10^, thermocapillary effect^11^, light-induced actuation^12,13^, and wire-guided droplet manipulation^14–16^. However, those methods either require complex fabrication process that increases their cost or suffer from the drawback that the surface eventually becomes contaminated, preventing their repeated use. Recently, magnetic actuation as an alternative mechanism for droplet manipulation has been of great research interest due to its remote, noninvasive manipulation ability and biocompatibility^17,18^. In the traditional magnetic manipulation method, paramagnetic particles or ferromagnetic particles are required to add into the droplets to serve as the actuator^19–26^, which limits the scope of its applications. To circumvent such a limitation, a new strategy is proposed to manipulate pure discrete droplets based on a magnetically actuated surface, which is usually created by impregnating a substrate with magnetic response elements (*e.g*., magnetic particles and steel balls). When a magnet is brought close to the surface, the surface deforms and creates a dent for droplet control. Seo *et al*. created a magnetic elastomer with a superhydrophobic surface for droplet manipulation, on which droplets can be moved by changing the surface topography using magnetic force^27^. Similarly, Sen *et al*. presented an approach for the manipulation of aqueous droplets on a soot-wax coated PDMS-iron particle composite substrate^28^. The surface profile around a droplet is altered due to magnetic force acting on the iron particles embedded in the substrate, which leads to droplet motion. Besides, Biswas *et al*. developed a new flexible magnet-actuated substrate by embedded individual steel balls into PDMS. When a steel ball is attracted to the permanent magnet below the surface, the flexible substrate is easily deformed to create a dent on the surface^29^. By deforming and shifting the location of the dent on the flexible substrate, one can easily control the movement of the droplet. In addition, magnetically actuating surfaces with micro- or nanoscale structures have considerable potential to be used for the active and dynamic manipulation of droplets because of their reversible and instantaneous structural tunability in response to the imposed magnetic field. For example, Kim *et al*. described a novel technique that can reversibly actuate a pure discrete droplet with only a permanent magnet by utilizing a magnetically responsive flexible film consisting of hierarchical pillars on the surface^30^. Wang *et al*. also fabricated a magnetically tunable rough surface based on nano hair-like structures grown on an array of micro-sized walls made of elastomers impregnated with magnetic particles. When a magnet pulled the wall, the droplet travelled toward the direction of pulling because the micro walls tilted towards the magnet^31^. In spite of avoiding the functionalization of droplets with magnetic particles, those magnetic actuation technologies for droplet manipulation require either fine-textured surfaces or complicated surface modifications that tend to increase their cost. Furthermore, all these methods can only manipulate droplets on a planar surface (*i.e*., in 2D). Manipulating droplets in 3D still represents a challenge.

Here, we propose a unique magnet-actuated platform for droplet manipulation, where a ferrofluid film floating on a liquid substrate serves as magnetic actuator. Due to the immiscibility of the oil-based ferrofluid and water, the droplets desired to be manipulated can stably rest on the surface of the floating ferrofluid film (FFF) under the action of surface tension, and a liquid-liquid interface is formed between the droplet and the FFF. The liquid-liquid interface leads to very low contact angle hysteresis and pinning-free droplet mobility, which is beneficial to fast droplet transportation and highly viscous droplet movement. In addition to serving as a force mediator, FFF can also be used as an encapsulation phase by wrapping a droplet and sinking into liquid substrate under the action of magnetic force. Based on this, novel functions, such as droplet encapsulation and controlled release of reactive cargo, can be realized with FFF-based platform. More importantly, in contrast to existing magnetic droplet manipulation methods, the FFF-based droplet manipulation can perform droplet operations in “full-space”, including droplet transport/coalescence above FFF (*i.e*. in air), droplet transport/coalescence on FFF and droplet encapsulation/release under FFF (*i.e*. in liquid), thus allowing complex tasks with droplets, and providing a versatile system for bioassays.

## Results

### FFF-based droplet manipulation platform

**Figure 1:**
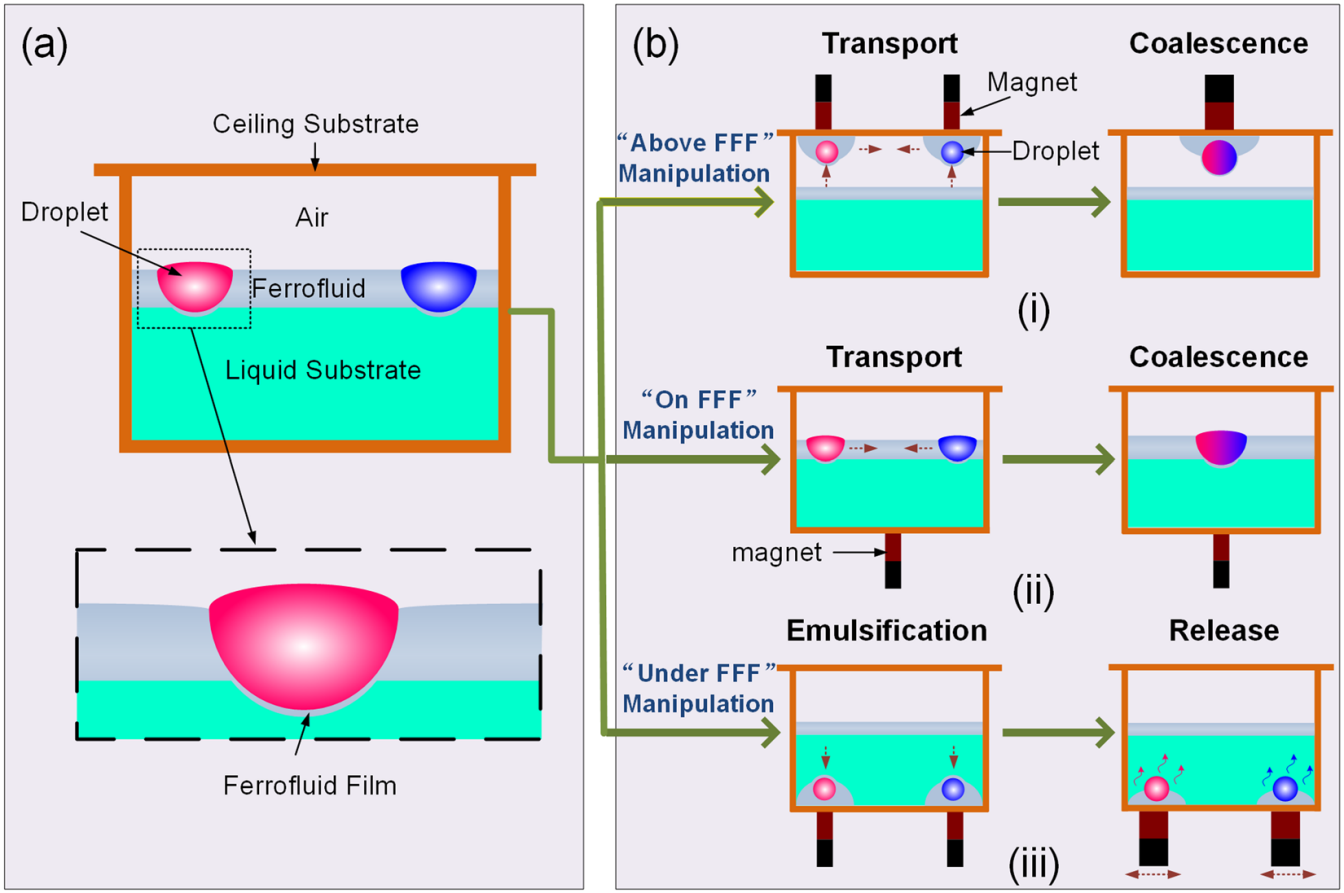
Schematic illustration of the FFF-based droplet manipulation platform. (**a**) The platform comprises three structural components: liquid substrate, floating ferrofluid film on the liquid substrate, and droplets on the floating ferrofluid film. (**b**) Illustration of the FFF-based droplet manipulation strategy: (i) droplet manipulation above FFF; (ii) droplet manipulation on FFF; (iii) droplet manipulation under FFF.

As illustrated in Fig. 1(a), our magnet-actuated droplet manipulation system comprises of a liquid substrate, a ferrofluid film floating on the surface of the liquid substrate and droplets settling on the FFF. Since the ferrofluid used in this study was oil-based, the water droplets stably float on top of it, thus forming a “no-stick” liquid-liquid interface. Due to the absence of direct contact between the liquid and the solid substrate, the liquid-liquid interface provides a low-friction surface for rapid and pinning-free moving of droplets. In contrast to lubricant-impregnated surfaces where an oil film is on a solid substrate^32,33^, FFF system containing a floating ferrofluid film on a liquid substrate offers extra degrees of freedom: the droplets settled on FFF can be enwrapped by ferrofluid and dragged upward to a ceiling substrate or dragged downward into the liquid substrate by magnetic force. Beyond the existing 2D magnet-actuated droplet manipulation, the FFF-based platform offers possibilities for magnetically-driven droplet manipulations in “full-space”, including above FFF, on FFF and under FFF (Fig. 1(b)).

### Droplets manipulation above FFF

**Figure 2:**
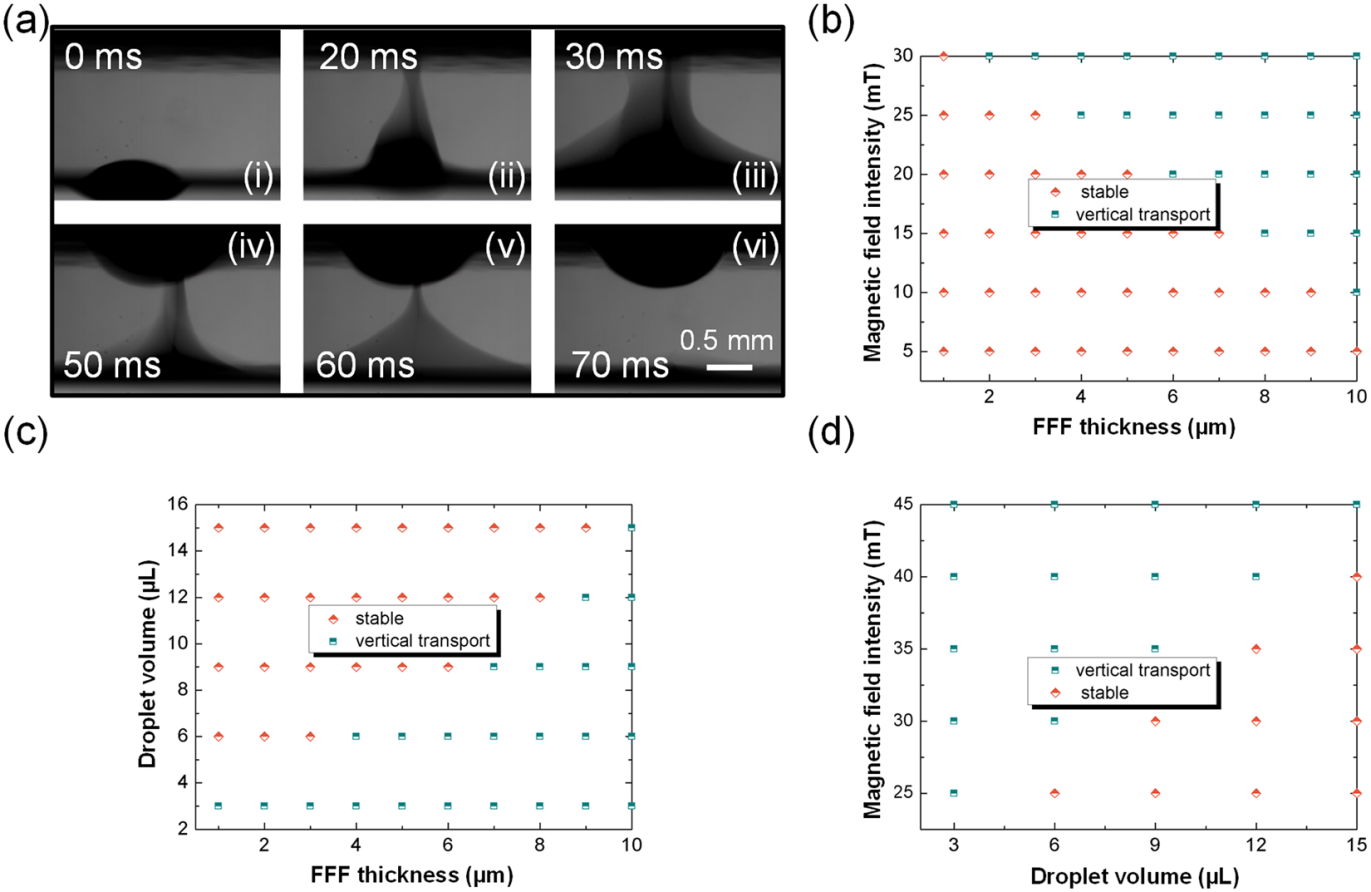
Droplets transport above FFF. (**a**) Consecutive images corresponding to droplets transport above FFF. (**b**) Droplet manipulation diagram with constant droplet volume (3 µL), and variations in magnetic field intensity and FFF thickness. (**c**) Droplet manipulation diagram with given magnetic field intensity (30 mT) and variations in droplet volume and FFF thickness. (**d**) Droplet manipulation diagram with a fixed FFF thickness (5 µm), and variations in droplet volume and magnetic field intensity.

As shown in Fig. 1(b)_(i), when a magnet bar vertically aligns with a droplet resting on the FFF and is lowered toward the ceiling substrate over the droplet, a part of FFF encapsulating the droplet is dragged upward and flew to the ceiling substrate under the action of magnetic force. Similar to droplet manipulation on the ferrofluid-impregnated surfaces, the hanging droplets on the ceiling substrate can be moved and merged by magnetic force. When horizontally moving the magnet, the ferrofluid drags the droplet, travelling on the surface of the ceiling substrate, and transferring the material to pre-designated locations. Coalescence can be realized by introducing a magnet centered between two neighbouring droplets. Figure 2(a) shows a series of images corresponding to vertical droplet transport above the FFF. Firstly, a 3 µL water droplet is added on the 5 µm FFF with the magnetic particles concentration of 30%. With applied magnetic field intensity of 25 mT, a part of FFF encapsulating the droplet is dragged upward and flew to the ceiling substrate within 70 ms. In view of this phenomenon, we recognize that droplet transport to the ceiling substrate is determined by the magnetic field intensity, FFF thickness and droplet volume. We therefore investigated the vertical droplet transport under different conditions. As shown in Fig. 2(b), with a fixed droplet volume (3 µL), vertical droplet transport mainly occurs at high magnetic field intensity and thick FFF, where the magnetic force acted on the FFF is large enough to overcome the gravitational force of the droplet to drag the entire droplet upward and flow to the ceiling substrate (see Supporting Information for detail discussion). The effect of droplet volume on droplet manipulation is examined by keeping the magnetic field intensity constant while changing FFF thickness and droplet volume (Fig. 2(c)). At a given magnetic field intensity (30 mT), droplet transport is observed with small droplets and thick FFF. Furthermore, the effect of magnetic field intensity on droplet operation is also analyzed. As shown in Fig. 2(d), with a fixed FFF thickness (5 µm), larger droplets need high magnetic field intensity to promote droplet transport. Consequently, it indicates that vertical droplet transport can be easily achieved just by balancing the three parameters of magnetic field intensity, FFF thickness and droplet volume.

**Figure 3:**
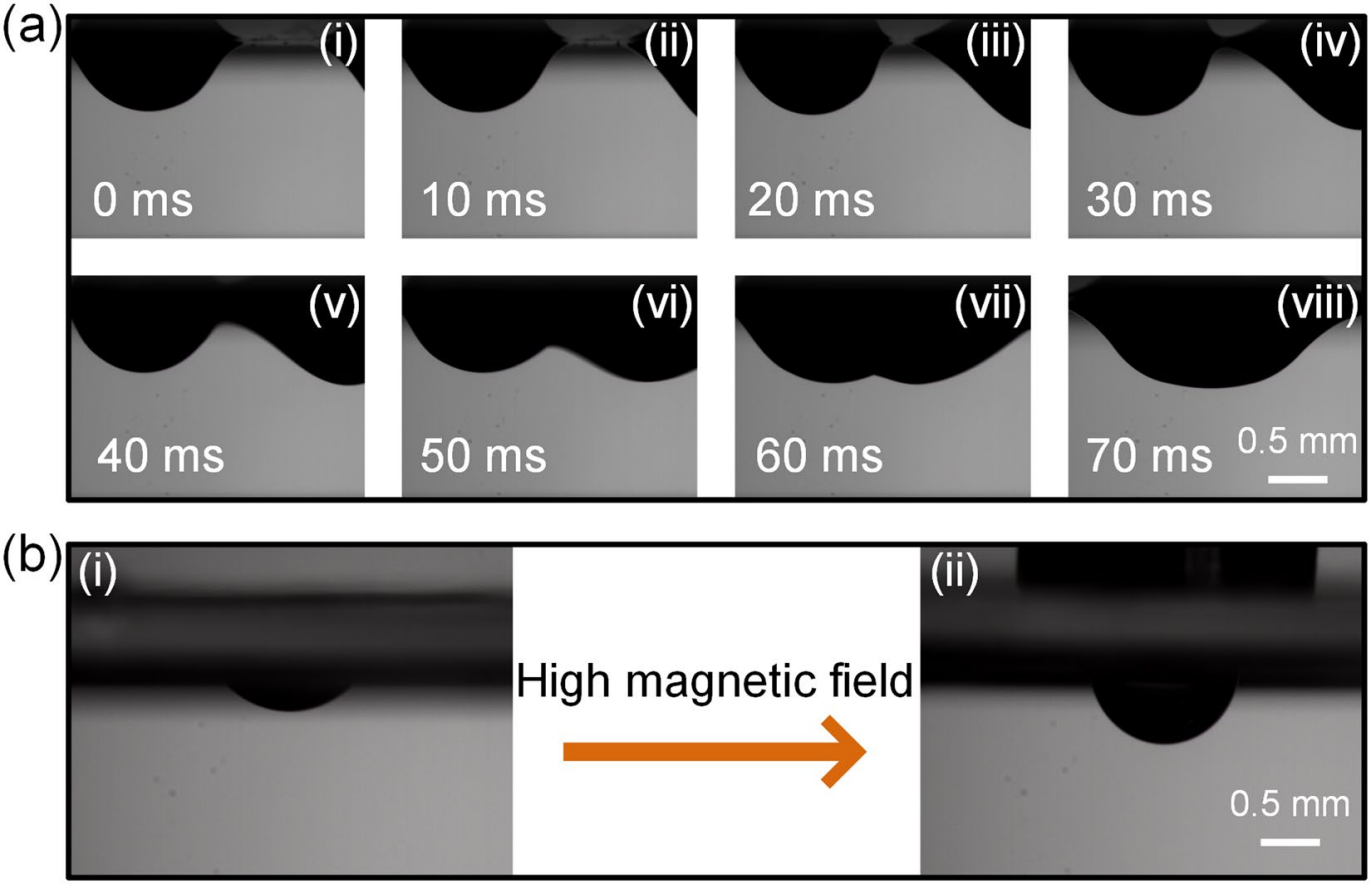
Droplets coalescence on the surface of the ceiling substrate. (**a**) Consecutive images corresponding to droplets coalescence on the surface of the ceiling substrate. (**b**) Deformation of the droplet covered by ferrofluid film as the applied magnetic field intensity increases.

Additionally, the coalescence of droplets was achieved on the ceiling substrate by simply introducing a magnet centered between two droplets (Fig. 3(a)). The region of highest magnetic field intensity, directly above the magnet, attracts both droplets to center under the magnet, leading to coalescence. As shown in Fig. 3(b), the ferrofluid will converge to the surface of the ceiling substrate when imposing a high magnetic field, which results in exposure of most area of water droplet enwrapped by ferrofluid. It is reasonable to suggest that two contact droplets on the ceiling substrate can easily coalesce after magnetically eliminating the barrier layer.

### Droplets manipulation on FFF

**Figure 4:**
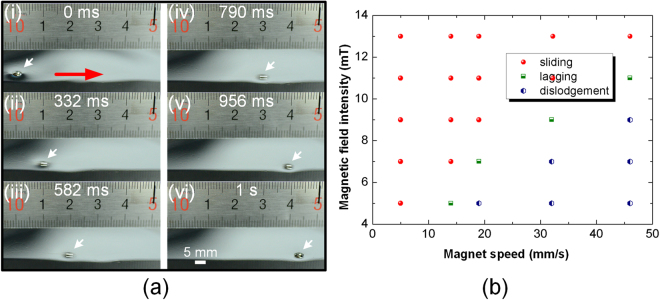
Droplet transport on FFF. (**a**) Snapshots of droplet transport on FFF. (**b**) Operating maps of the floating water droplet transport on FFF with different magnet speed and magnetic field intensity.

Similar to other magnet-actuated methods, the FFF-based platform can also perform droplet manipulations on 2D liquid surface by simply placing a magnet beneath the liquid substrate (Fig. 1(b)_(ii)). Due to the low friction of the liquid-liquid interface, droplets can move quickly and smoothly with an external magnet. Figure 4(a) shows the images corresponding to droplets transport on the FFF. With a magnetic field intensity (14 mT), a 10 µL droplet slides with the magnet with velocity of 46 mm/s on the 5 µm ferrofluid film with the magnetic particles concentration of 30%. The direction and the speed of the droplet motion are easily controlled by the motion of magnets with low magnetic field intensity. Figure 4(b) shows the operating maps of the floating water droplet on the ferrofluid film of 5 µm in corresponding to different magnet speed and magnetic field intensity with a constant of droplet volume (10 µL). Three phenomena are observed: sliding, lagging and dislodgment. With high magnetic field intensity and slow magnet movement, the droplet slides following the magnet to with the same velocity as that of the magnet. At a given magnetic field intensity, when the magnet speed exceeds a critical value, the droplet will lag behind, resulting in its dislodgement from the influence of the magnet. The lagging and dislodging behaviors are mainly due to the high friction force caused by increasing the speed of the magnet (see Supporting Information for detail discussion). Obviously, a higher magnetic field intensity shows better control of the movement of droplet when the magnet speed increases. Therefore, the maximum speed of a droplet floating on the ferrofluid film mainly depends on the magnet speed and magnetic field intensity.

**Figure 5:**
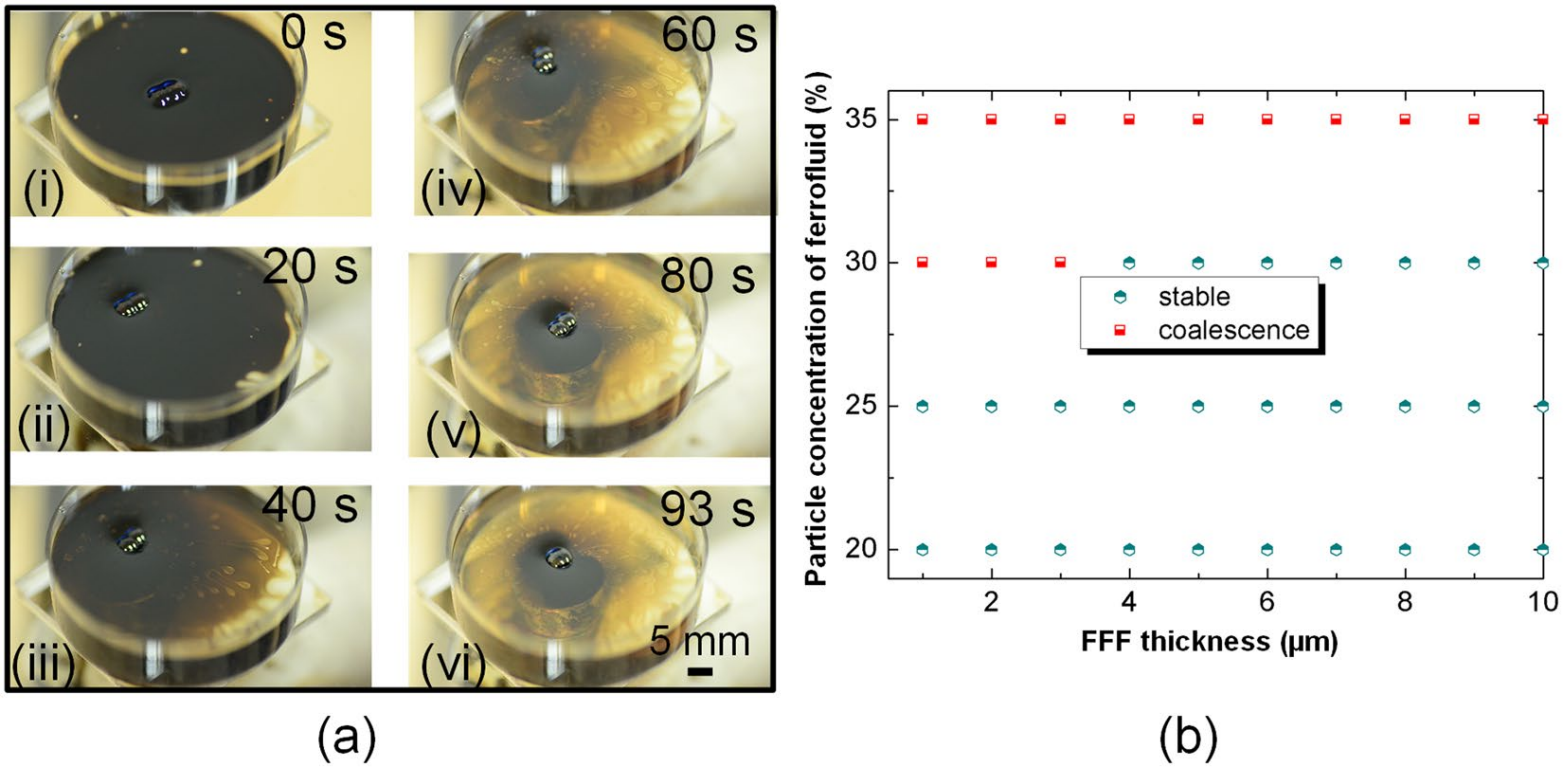
Droplets coalescence on FFF. (**a**) Snapshots of droplets coalescence on FFF. (**b**) Operating diagram of the droplets coalescence on FFF with different particle concentration of the ferrofluid.

Similarly, two droplets on FFF can be brought into contact and then fuse together with the aid of the magnetic field (Fig. 1(b)_(ii)). Figure 5(a) shows the images corresponding to droplets coalescence on the FFF. When two water droplets came into contact on the oil-based FFF, they exhibited noncoalescence due to the formation of a thin oil-based ferrofluid film between the two droplets. After applying a moderate magnetic field (25 mT) beneath the liquid substrate last for 93 s, coalescence between two droplets in contact can be realized since the magnetic force thins the ferrofluid film surrounding the droplets. However, due to the mobility and deformability of liquid substrate, the thinning effect of magnetic force on the ferrofluid film enwrapping the droplets is less effective. Thus, coalescence of droplets on the FFF requires more time than that on the ceiling substrate. We also investigated the conditions for the droplets coalescence on the FFF. From Fig. 5(b), it can be found that droplets coalescence on the FFF mainly depends on the magnetic particles concentration of the ferrofluid. High particle concentration of the ferrofluid is favorable for coalescing droplets on FFF. Since the magnetic particles carry more oil toward magnet which leads to a thinner oil film surrounding the droplets so that it is not sufficient to prevent coalescence.

### Droplets manipulation under water

**Figure 6:**
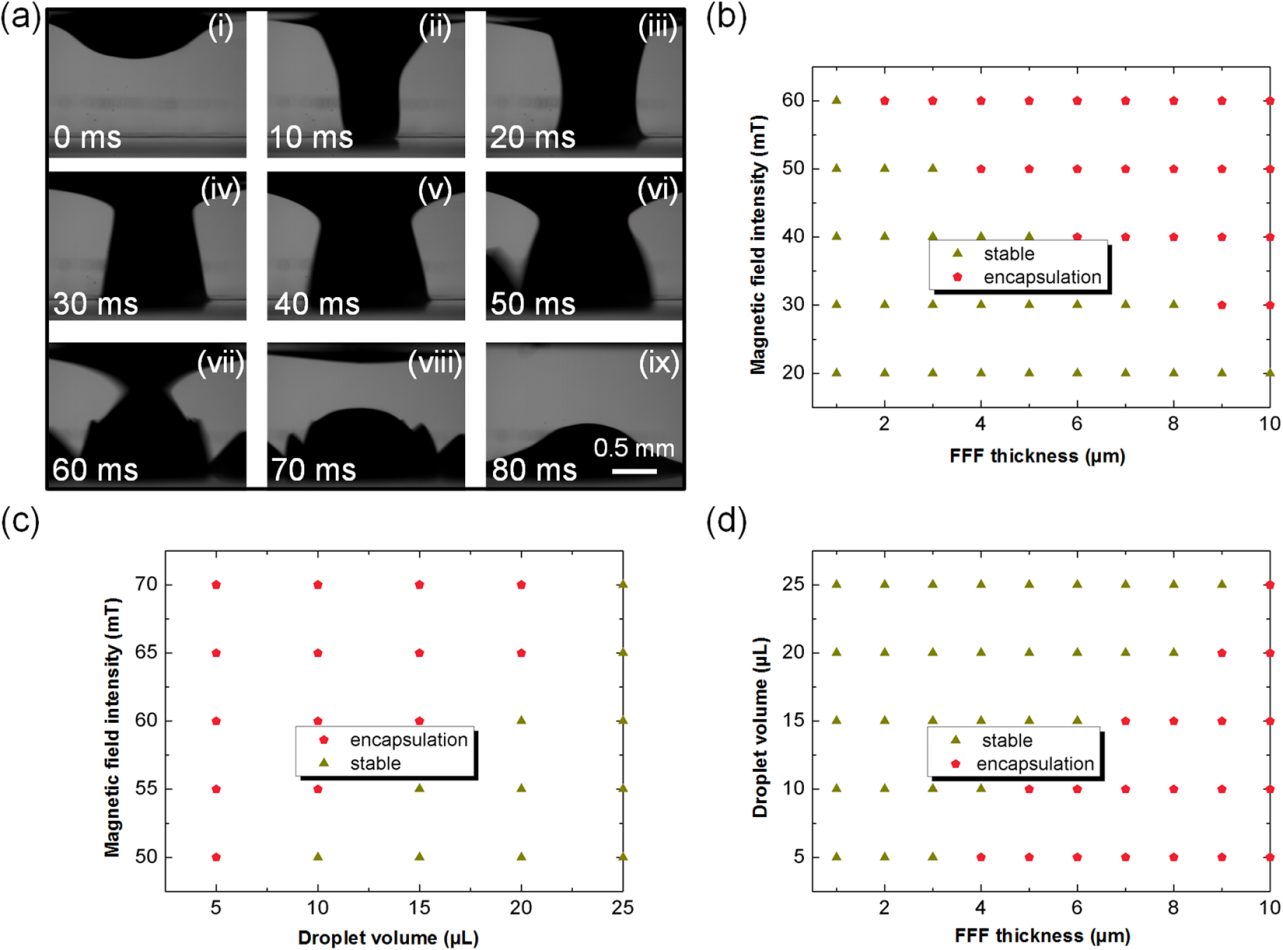
Droplets encapsulation under water. (**a**) Consecutive images corresponding to droplets encapsulation processes under water. (**b**) Droplet encapsulation diagram with constant droplet volume (5 µL), and variations in magnetic field intensity and FFF thickness. (**c**) Droplet encapsulation diagram with a fixed FFF thickness (5 µm), andvariations in droplet volume and magnetic field intensity. (**d**) Droplet encapsulation diagram with given magnetic field intensity (55 mT) and variations in droplet volume and FFF thickness.

Interestingly, the FFF-based platform can perform droplet manipulations under water. By applying a high intensity magnet beneath the liquid substrate, the droplets can be encapsulated by the FFF and sink into the liquid substrate, which provides a simple and convenient droplet emulsification method (Fig. 1(b)_(iii)). Figure 6(a) shows the images corresponding to droplets encapsulation processes under water. Firstly, a 5 µL water droplet is floating on the 5 µm ferrofluid film with the magnetic particles concentration of 30%. By applying magnetic field intensity of 50 mT beneath the liquid substrate, the droplets can be encapsulated by the FFF and fallen to the bottom of the water within 80 ms. Therefore, this magnet-assisted droplet encapsulation provides a quick and convenient droplet emulsification method. To make better use of the magnet-assisted droplet encapsulation, we also investigated the droplet encapsulation under different conditions, including the magnetic field intensity, FFF thickness and droplet volume. As shown in Fig. 6(b), with a fixed droplet volume (5 µL), droplet encapsulation occurs at high magnetic intensity and thick FFF. Under these conditions, the net force of the gravitational force of the droplet and the magnetic force acted on the ferrofluid film is large enough to overcome to capillary force and the buoyancy force induced by the liquid substrate (see Supporting Information for detail discussion). Furthermore, the effect of magnetic field intensity on droplet encapsulation is also analyzed. As shown in Fig. 6(c), with a fixed film thickness (5 µm), larger droplets require high magnetic field intensity to accomplish droplet encapsulation process. Besides, droplet volume on droplet operation is examined by keeping the magnetic field intensity constant while changing FFF thickness and droplet volume (Fig. 6(d)). At a given magnetic field intensity (55 mT), droplet transport is observed with small droplets and high film thickness. Thus, droplet encapsulation can be easily achieved with suitable conditions among magnetic field intensity, FFF thickness and droplet volume.

**Figure 7:**
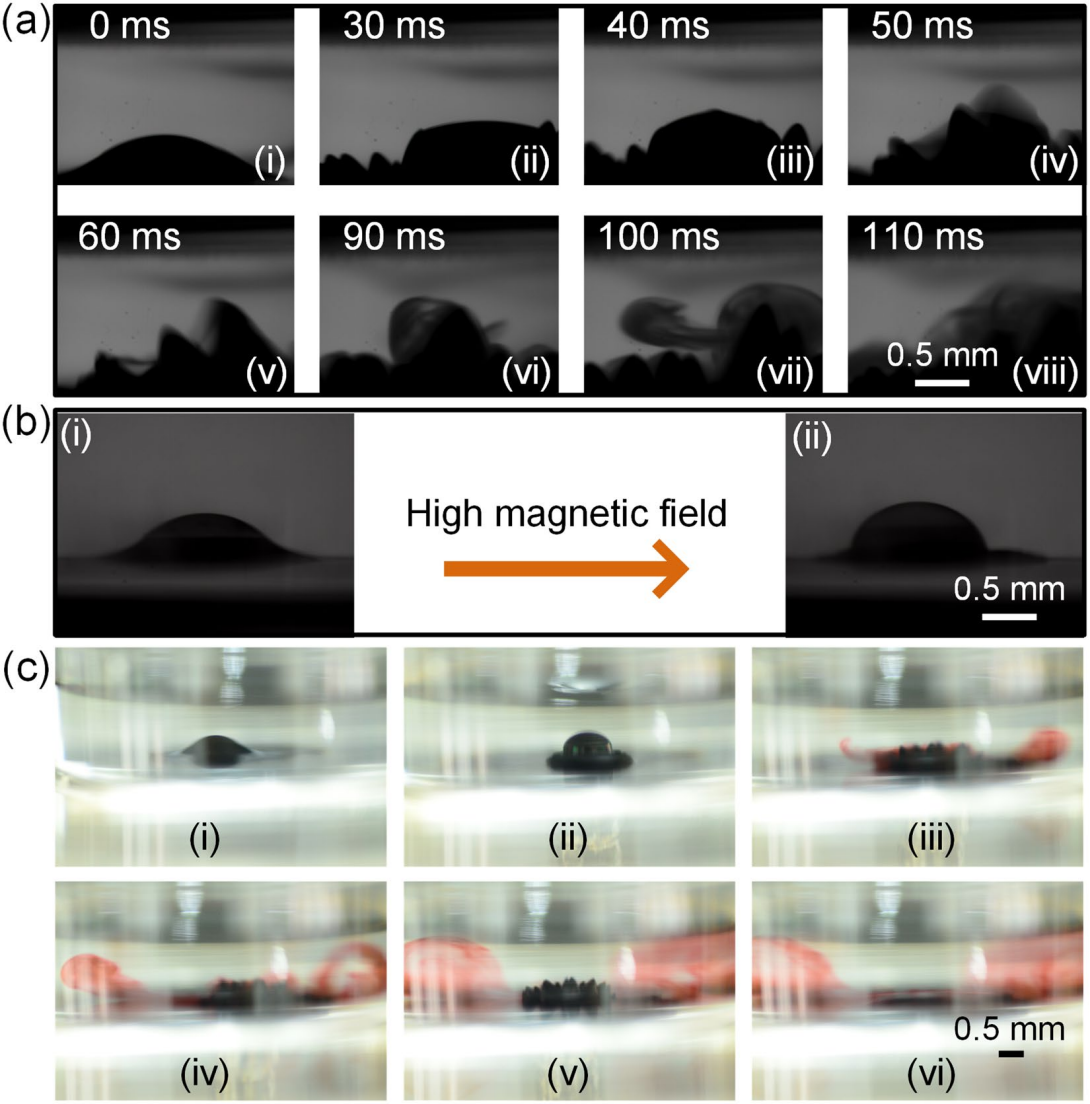
The release process of an encapsulated droplet under water. (**a**) Snapshots of the release process of an encapsulated droplet under water. (**b**) Deformation ofthe droplet covered by ferrofluid film as the applied magnetic field intensity increases. (**c**) Snapshots of the release process of an encapsulated droplet containing dye.

After the completion of ferrofluid encapsulation, the emulsification droplets can transport to the anywhere under water with the magnetic field intensity of 50 mT. As shown in Fig. 7(a), when the encapsulated droplets transport to the target position, it can be released on demand by applying a high magnetic field (250 mT) within 50 ms. The horizontal oscillation of the magnet can accelerate the decomposition process. As shown in Fig. 7(b), with the imposed high magnetic field, the water droplet can be pulled out of the ferrofluid shell because the vast majority of the ferrofluid film will converge to the bottom of the petri dish. Thus, the covering ferrofluid film on the surface of the droplet becomes extremely thin. Owing to the water pressure imposed on the surface of the droplet, breakup of the droplets can be immediately realized (Fig. 7(c)). The materials (food dyes) in the droplets is completely released into the liquid substrate.

### Maximum droplet loading volume on the FFF

**Figure 8:**
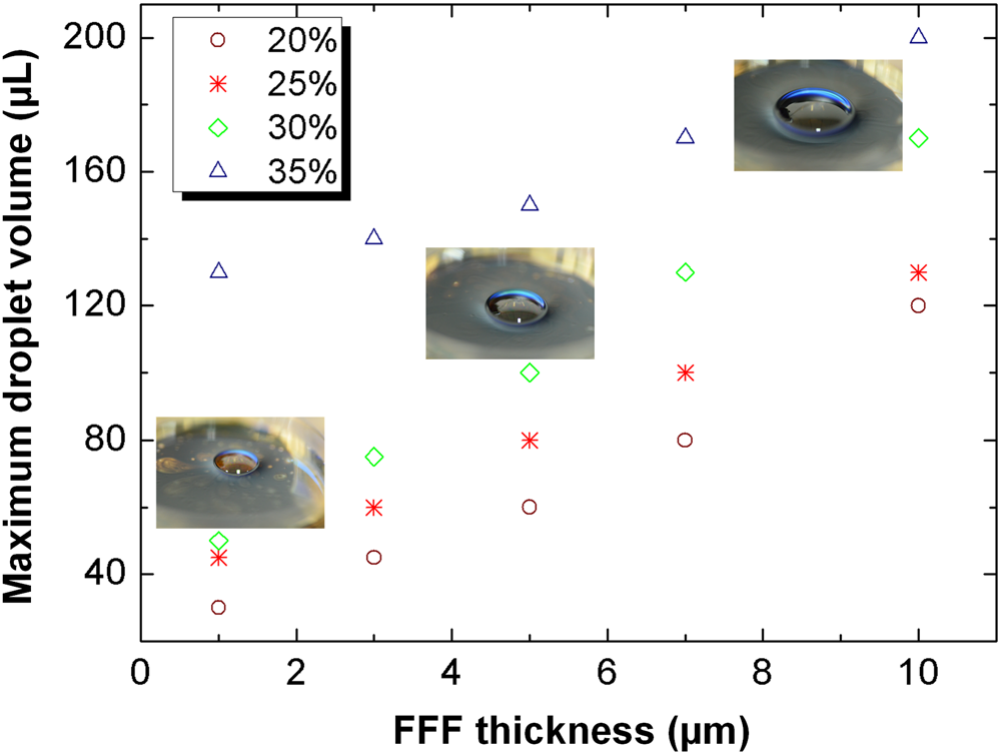
The relationship between the FFF thickness and the maximum droplet loading volume with different particle concentration of the ferrofluid.

From above, we know that the FFF can bear the weight of small droplets. To demonstrate the feasibility of FFF for transporting large droplets, we quantitatively investigate the relationship between the FFF thickness and the maximum droplet loading volume with different particle concentration of the ferrofluid. As shown in Fig. 8, with the increase of FFF thickness, the maximum droplet loading volume increases. Besides, at the same FFF thickness, the maximum droplet loading volume increases with increasing the particle concentration of the ferrofluid due to the increased viscosity of the FFF. The embedded pictures in the Fig. 8 represent the maximum droplet loading volume of 50 µL, 100 µL, and 170 µL at the constant particle concentration of 30% with the film thickness of 1 µm, 5 µm, and 10 µm, respectively. The maximum droplet loading volume can reach 200 µL with particle concentration of 35% at 10 µm. It indicates that FFF allows manipulate droplets in a greater volume range.

## Discussion

We have presented a novel method to manipulate free aqueous microdroplets by using a floating ferrofluid film. With the aid of the liquid substrate, the FFF-based platform provides extra degrees of freedom for more versatile liquid handling, especially allowing to conveniently manipulate droplets in “full-space” that traditional magnetic droplet platforms cannot handle. Benefited from its non-stick liquid-liquid interface, the FFF-based platform is able to manipulate a variety of different liquids, including diamagnetic and viscous, greatly expand the scope of fluids handled by magnet-actuated methods. Basic manipulation of droplets was demonstrated, which involved transporting, merging, encapsulating and releasing of droplets with FFF-based platform. We expect that the suggested method of droplet manipulation can open up new possibilities for magnetic digital microfluidics.

## Methods

### Experimental Setup

The experimental setup built for studying FFF-based droplet manipulation consisted of a tripod, a petri dish, a permanent magnet and a programmable x-y-z stage. The petri dish (35 mm in diameter) was used to hold the supporting water, which was purchased from Health One Co., Ltd, China. In particular, during the measurement of droplet transport speed experiment, we will replace the 35 mm petri dish with 90 mm in diameter for large droplet transport space. The petri dish is placed on the tripod, which is purchased from Boshirong Co., Ltd, China. The cylinder permanent magnet (Neodymium, 12 mm in diameter) was purchased from Ruifeng Magnet Co., Ltd, China. It was used as a “hand” to manipulate droplet, which was mounted on a automatically programmable 3D stage. Oil-based ferrofluid with different magnetic particle concentrations was purchased from Song Inc, Japan (oil-based Kt series), which shows unique magnetic and oil properties to meet the requirements of our droplet manipulaiton (see Supporting Information for detailed properties of the ferrofluid).

### Instruments and Characterizations

The magnetic flux density of the magnet was measured with a handheld commercial gaussmeter (SJ200, measurement range from 0 to 2 T, Jason Technology Co., Ltd, China). Dynamic behaviors of the droplets were consecutively recorded with by a high-speed CCD camera (Shanghai FangRui Instrument Co., Ltd, China). A digital camera (D7000, Nikon, Japan) was used to captured the photograph of the droplet manipulation process.

### Estimation of FFF thickness

First, water (5 mL) was added into the petri dish using injection syringe, which acted as the liquid substrate. Then, ferrofluid was added on the surface of the liquid substrate using pipettor to form FFF. Assuming that the ferrofluid was even floating on the surface of the water, the thickness of the FFF was calculated based on the added ferrofluid volume divided by the top area of the liquid substrate.

## Electronic supplementary material

Supporting Information

## References

[1] Cho SK, Moon HJ, Kim CJ (2003). Creating, transporting, cutting, and merging liquid droplets by electrowetting-based actuation for digital microfluidic circuits. J. Microelectromech. Syst..

[2] Srinivasan V, Pamula VK, Fair RB (2004). An integrated digital microfluidic lab-on-a-chip for clinical diagnostics on human physiological fluids. Lab Chip..

[3] Fair RB (2007). Digital microfluidics: is a true lab-on-a-chip possible?. Microfluid. Nanofluid..

[4] Sista R (2008). Development of a digital microfluidic platform for point of care testing. Lab Chip..

[5] Barbulovic-Nad I, Au SH, Wheeler AR (2010). A microfluidic platform for complete mammalian cell culture. Lab Chip..

[6] Guttenberg Z (2005). A. Planar chip device for PCR and hybridization with surface acoustic wave pump. Lab Chip..

[7] Beyssen D, Brizoual L, Le., Elmazria O, Alnot P (2006). Microfluidic device based on surface acoustic wave. Sens. Actuators B: Chem..

[8] Renaudin A, Tabourier P, Camart J-C, Druon C (2006). Surface acoustic wave two-dimensional transport and location of microdropletsusing echo signal. J. Appl. Phys..

[9] Ding X (2013). Surface acoustic wave microfluidics. Lab Chip..

[10] Huang CJ, Fang WF, Ke MS, Chou HYE, Yang JT (2014). A biocompatible open-surface droplet manipulation platform for detection of multi-nucleotide polymorphism. Lab Chip..

[11] Darhuber AA, Valentino JP, Davis JMS, Troian M, Wagner S (2003). Microfluidic actuation by modulation of surface stresses. Appl. Phys. Lett..

[12] Park S, Chiou PY (2011). Light-driven droplet manipulation technologies for lab-on-a-chip applications. Adv. OptoElectron..

[13] Baigl D (2012). Photo-actuation of liquids for light-driven microfluidics: state of the art and perspectives. Lab Chip..

[14] Yoon J-Y, You DJ (2008). Backscattering particle immunoassays in wire-guide droplet manipulations. J. Biol. Eng..

[15] Harshman DK (2014). Enhanced nucleic acid amplification with blood *in situ* by wire-guided droplet manipulation (WDM). Biosens. Bioelectron..

[16] Zhang Y, Wittstock G (2017). A platform for electric field aided and wire-guided droplet manipulation. Small..

[17] Zhang Y, Nguyen N-T (2017). Magnetic digital microfluidics-a review. Lab Chip..

[18] Huang G (2017). Magnetically actuated droplet manipulation and its potential biomedical applications. ACS Appl. Mater. Interfaces..

[19] Ohashi T, Kuyama H, Hanafusa N, Togawa Y (2007). A simple device using magnetic transportation for droplet-based PCR. Biomed. Microdevices..

[20] Schneider J (2008). Motion of viscous drops on superhydrophobic surfaces due to magnetic gradients. Colloids Surf..

[21] Zhang Y, Wang T-H (2013). Full-range magnetic manipulation of droplets via surface energy traps enables complex bioassays. Adv. Mater..

[22] Lehmann U, Vandevyver C, Parashar VK, Gijs MAM (2006). Droplet-based DNA purification in a magnetic lab-on-a-Chip. Angew. Chem. Int. Ed..

[23] Lehmann U (2007). On-chip antibody handling and colorimetric detection in a magnetic droplet manipulation system. Microelectron. Eng..

[24] Lehmann U (2006). Two-dimensional magnetic manipulation of microdroplets on a chip as a platform for bioanalytical applications. Sens. Actuators B: Chem..

[25] Long Z, Shetty AM, Solomon MJ, Larson RG (2009). Fundamentals of magnet-actuated droplet manipulation on an open hydrophobic surface. Lab Chip..

[26] Mats L, Young R, Gibson GTT, Oleschuk RD (2015). Magnetic droplet actuation on natural (Colocasia leaf) and fluorinated silica nanoparticle superhydrophobicsurfaces. Sens. Actuators, B..

[27] Seo KS, Wi R, Im SG, Kim DH (2013). A superhydrophobic magnetic elastomer actuator for droplet motion control,Polym. Adv. Technol..

[28] Damodara S, Sen AK (2017). Magnetic field assisted droplet manipulation on a soot-wax coated superhydrophobic surface of a PDMS-iron particle composite substrate. Sens. Actuators, B..

[29] Biswas S, Pomeau Y, Chaudhury MK (2016). New drop fluidics enabled by magnetic-field-mediated elastocapillary transduction. Langmuir..

[30] Kim JH (2015). Remote manipulation of droplets on a flexible magnetically responsive film. Sci Rep..

[31] Wang L (2015). Dynamic magnetic responsive wall array with droplet shedding-off properties. Sci. Rep..

[32] Smith JD (2013). Droplet mobility on lubricant-impregnated surfaces. Soft Matter..

[33] Khalil KS, Mahmoudi SR, Abu-dheir N, Varanasi KK (2014). Active surfaces: Ferrofluid-impregnated surfaces for active manipulation of droplets. Appl. Phys. Lett..

