# Supplementary material for: A novel magnet-actuated droplet manipulation platform using a floating ferrofluid film
Source: Sci Rep. 2017 Nov 16;7:15705. doi: 10.1038/s41598-017-15964-8 (PMC5691075; doi:10.1038/s41598-017-15964-8)
Supplement: Supplementary file 1 — Supporting Information [file 41598_2017_15964_MOESM1_ESM.pdf]

Supporting Information

Title: A novel magnet actuated droplet manipulation platform using a floating ferrofluid film

Chao Yang & Gang Li*

Defense Key Disciplines Lab of Novel Micro-Nano Devices and System Technology, Key Laboratory of Optoelectronic Technology and Systems, Ministry of Education, Chongqing University, Chongqing 400044, China

*Correspondence and requests for materials should be addressed to G. Li. (email: gang_li@cqu.edu.cn)

Table S1. Property of the ferrofluid.

Ferrofluid type	Kt-0314	Kt-0452	Kt-0533	Kt-0685
Magnetic particles concentrations	20%	25%	30%	35%
Density(kg/m^3)$\times 10^3$	1.18	1.23	1.30	1.32

Viscosity(cp)(25℃)	8	20	100	200
Saturationmagnetization(GS)	200±20	450±50	450±50	450±50
Initialsusceptibility(m/H)	0.6	0.8	0.8	0.8

Physical Model for Droplet Manipulation

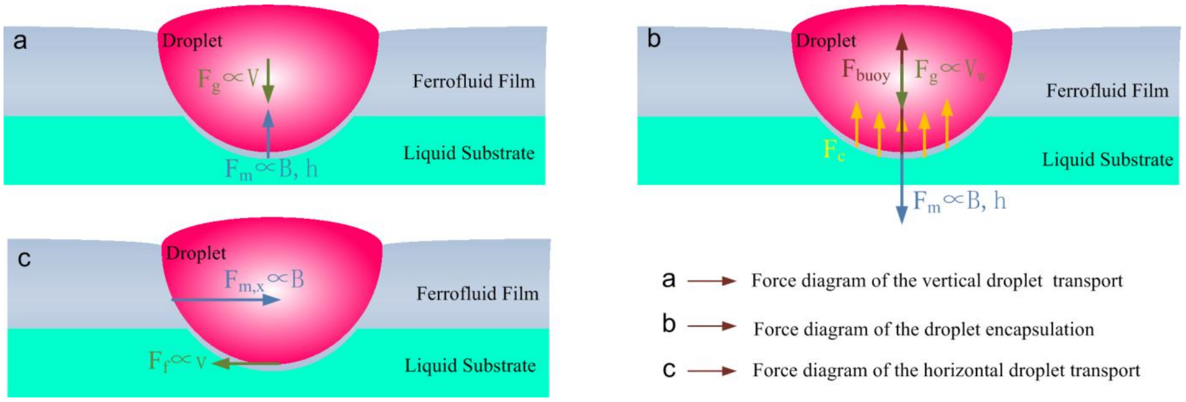

Figure S1. Physical model for different droplet manipulation processes.

The magnetic force acting on the floating ferrofluid film (FFF) is estimated as[S1]

$$F_m = \mu_0 \chi \nabla H^2 V . \tag{1}$$

Here, V is volume of the FFF, χ is the magnetic susceptibility of the ferrofluid, H is the magnetic field and$\mu_0$denotes vacuum magnetic permeability. The magnetic field H in the Equation 1 is expressed as,

$$H = B\mu_0^{-1}(1+\chi)^{-1} . \tag{2}$$

Here, B denotes induction magnetic field measured with the gaussmeter in the experiment. In our experiment, the volume of the FFF can be calculated by,

$$V = \pi r^2 h . \tag{3}$$

Here, h is the thickness of the FFF, r is the radius of the petri dish. Since other parameters are fixed, Equation 1 can be further simplified as,

$$F_m \propto B, h . \tag{4}$$

The gravitational force of the droplet is given by,

$$F_g = \rho_w V_w g \,. \tag{5}$$

Here, V_w is the volume of the droplet, ρ_w is the mass density of the water, and g is the acceleration of gravity, respectively.

As shown in Fig. S1(a), along the operating boundary of the droplet vertical transport region, we have,

$$F_m = F_g \,. \tag{6}$$

Thus, substituting from Equation (1) and (5), we get,

$$\nabla B^2 = \frac{\rho_W V_w g \mu_0 (1+\chi)^2}{\pi r^2 h \chi} \,. \tag{7}$$

Therefore, the applied magnetic field for droplet vertical transport depends on the droplet volume and the FFF thickness.

When the droplet is encapsulated by the FFF under the action of magnetic force and sinks into the liquid substrate, it will press against the liquid substrate. Thus, the capillary force can be written as[S2],

$$F_c = 2\pi R \gamma_{w-f} \,. \tag{8}$$

Here, $\gamma_{w\text{-}f}$ is the interface tension between water and oil-based ferrofluid, and R is the radius of the bottom contact area between the ferrofluid encapsulation droplet and the supporting water. Besides, the buoyancy force induced by the water substrate also acts against it, it gives,

$$F_{buoy} = \rho_w V_w g + \rho_w \pi r^2 h g \,. \tag{9}$$

As shown in Fig. S1(b), to simplify the equation for scaling analysis, the force balance in the droplet encapsulation region is

$$F_m + F_g = F_c + F_{buoy} \,. \tag{10}$$

Thus, substituting from Equation (1), (5), (8), and (9), we get,

$$\nabla B^2 = \frac{(6^{\frac{1}{3}} \pi^{\frac{2}{3}} V_w^{\frac{1}{3}} \gamma_{w-f} + \rho_w \pi r^2 h g) \mu_0 (1+\chi)^2}{\pi r^2 h \chi} \,. \tag{11}$$

Therefore, the applied magnetic field for droplet encapsulation also depends on the droplet volume and the thickness of the FFF.

As shown in Fig. S1(c), when droplet transport over the FFF, the frictional force of the droplet is given by [S3],

$$F_f \cong K_f R \mu_f v \,. \qquad (12)$$

Here, K_f is the friction constant, μ_f isthe viscosity of the ferrofluid and v is the velocity of the drop. Thus, the scaling relationship between friction force and speed is simplified as,

$$F_f \propto v \,. \qquad (13)$$

The relationship indicates that increasing the speed of the magnet will increase the friction force causing the droplet to lag behind the moving permanent magnet and eventually to dislodge.

References

S1. Khalil, K.S., Mahmoudi, S. R., Abu-dheir, N. & Varanasi, K. K. Active surfaces: Ferrofluid-impregnated surfaces for active manipulation of droplets. *Appl. Phys. Lett.* **105,** 041604 (2014).
S2. Zuo, P., Liu, J. & Li, S. The load-bearing ability of a particle raft under the transversecompression of a slender rod. *Soft Matter.* **13,** 2315 (2017).
S3. Khaw, M. K., Ooi, C. H., Mohd-Yasin, F., Vadivelu, R., John ,J. S. & Nguyen, N.-T. Digital microfluidics with a magnetically actuated floating liquid marble. *Lab Chip.* **16**, 2211-2218 (2016).
